# Behavioral and dietary determinants of central adiposity assessed by ABSI in a mediterranean clinical sample

**DOI:** 10.1017/S1368980025101729

**Published:** 2025-12-26

**Authors:** Mauro Lombardo, Jesse C. Krakauer, Nir Y. Krakauer

**Affiliations:** 1 Department for the Promotion of Human Science and Quality of Life, San Raffaele Open University, Rome, Italy; 2 Corewell Health William Beaumont University Hospital, Royal Oak, MI, USA; 3 Department of Civil Engineering, City College of New York, New York, NY, USA

**Keywords:** ABSI, Abdominal adiposity, Mediterranean diet, Eating behaviour, Physical activity

## Abstract

**Objective:**

A Body Shape Index (ABSI) is a validated anthropometric measure describing body shape independently of BMI and height. This study aimed to evaluate the association between ABSI and dietary quality and eating behaviours in a Mediterranean clinical population.

**Design:**

We conducted a cross-sectional study analysing associations between ABSI and diet/behaviour using Pearson correlations and multivariable linear regressions adjusted for age, sex and BMI.

**Setting:**

The study took place at a Mediterranean diet-based nutrition clinic in Rome, Italy.

**Participants:**

The sample included 1640 adult patients attending follow-up visits at the clinic. ABSI *z*-scores were calculated and standardised by age and sex. Weekly food intake was assessed using 7-day food diaries, and behavioural preferences were collected via structured questionnaires.

**Results:**

The Pearson correlation between BMI and internal *z*-scored ABSI (zABSI) was weak but statistically significant (*r* = 0·113, *P* < 0·0001), confirming that ABSI captures body shape independently from BMI. As expected, ABSI strongly correlated with WC (*r* = 0·78, *P* < 0·001). Playing a sport was inversely associated with zABSI (*β* = –0·365, *P* < 0·001). Nighttime eating (*β* = 0·237, *P* = 0·001), snacking between meals (*β* = 0·133, *P* = 0·014) and preference for sweet over salty foods (*β* = 0·025, *P* = 0·010) were positively associated with higher ABSI values.

**Conclusions:**

In this Mediterranean clinical sample, ABSI identified behavioural and dietary correlates of body shape-related risk. Promoting physical activity and addressing nighttime eating may help improve anthropometric profiles linked to abdominal fat distribution.

## Graphical abstract

**Figure:**
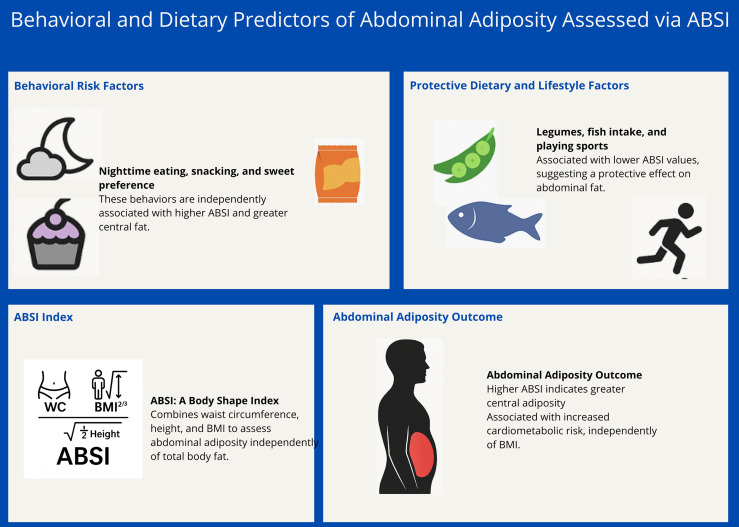

Body composition and fat distribution are recognised as determinants of cardiometabolic risk. The BMI is the most commonly used anthropometric measure, but it does not distinguish between fat and lean mass or account for fat distribution. To overcome these limitations, the A Body Shape Index (ABSI) was developed, an allometric index that standardises waist circumference (WC) for height and weight, providing an index of body shape and central fat distribution independent of BMI. The ABSI has demonstrated predictive value for mortality, cardiovascular events and metabolic outcomes in various populations^(1–4)^.

Diet plays a central role in modulating adiposity and metabolic health. Numerous studies have examined individual foods or nutrients, but the association between overall dietary profiles and body shape, as described by ABSI, remains insufficiently explored^(5)^. Composite indices such as the Mediterranean diet score or plant-based dietary indices have been associated with better metabolic outcomes, but their relationship with ABSI has not been extensively studied^(6)^. In addition to diet composition, dietary behaviours may also contribute to fat distribution. Practices such as skipping meals, eating fast or eating out often have been linked to negative metabolic effects^(7)^, but their potential relationship with ABSI is unclear.

In this study, we analysed data from adult patients who were prescribed a Mediterranean-type diet. During the first follow-up visit, 7-day food diaries and structured questionnaires were collected to assess food intake and eating behaviour. We tested the association between ABSI and specific food groups and composite dietary and behavioural indices. We hypothesised that healthier and more sustainable eating patterns were inversely associated with abdominal adiposity as measured by ABSI.

## Methods

### Study design and participants

This cross-sectional study was conducted in a specialised medical centre in Rome, Italy, between January 2023 and November 2024. It involved adult patients (age ≥18 years) who were prescribed a Mediterranean-type diet. Anthropometric measurements, including weight, height and WC used to compute ABSI, were collected at the initial clinical visit, before starting the prescribed diet. Participants then followed the Mediterranean plan and maintained remote contact with the clinical team through a dedicated chat system for clarification and support. After approximately 3–4 weeks, they returned for a follow-up visit, during which the 7-day food diaries and behavioural questionnaires were collected.

Inclusion criteria required participants to be fluent in Italian and able to complete a structured food diary and questionnaire. Written informed consent was obtained from all participants. All participants received standardised educational sessions on the Mediterranean diet at baseline. These sessions, conducted by registered dietitians, included detailed instructions on portion sizes, food groups and practical strategies to improve adherence. Visual aids and standardised portion references were provided to support the accuracy of the data in the food diaries. Exclusion criteria included incomplete or missing anthropometric data or food diaries, implausible body composition values and diagnosed metabolic conditions that could influence eating habits or body fat distribution. Subjects on strict therapeutic diets or using meal replacements were also excluded. The study was approved by the ethics committee of the Lazio area 5 territorial ethics committee (approval code: N.57/SR/23; approval date: 7 November 2023) and registered on ClinicalTrials.gov (NCT06654674).

A total of 1810 participants were initially screened. Of these, 62 (3·4 %) were excluded for missing anthropometric data, 54 (3·0 %) for incomplete or missing 7-day food diaries, and 37 (2·0 %) for implausible body composition values (e.g. BMI > 60 kg/m^2^, negative fat-free mass). An additional 17 (0·9 %) subjects were excluded due to ongoing therapeutic or replacement diets that could bias dietary assessment. The final analytic sample therefore included 1640 participants. Missing values among behavioural questionnaire items were low (<3 % across variables) and handled through listwise deletion. No imputation procedures were applied to maintain transparency and analytical consistency. Clinical records also contained information on comorbidities such as diabetes, metabolic syndrome and hypertension. However, these data were not included in the analytic database, as the study focused on behavioural and dietary correlates of abdominal adiposity.

### Evaluation of the diet

Dietary data were collected using 7-day food diaries completed by participants at home after receiving nutrition education. The 7-day food diaries used in this study have previously shown good agreement with objective biomarkers and are considered a valid tool for assessing habitual food intake in clinical populations^(8)^. The diaries focused on the frequency of consumption of major food groups, including meat, processed meat, fish, eggs, dairy products, legumes and soybeans. Registered dietitians reviewed the diaries and manually counted the total number of weekly servings for each food group. Participants could choose from isocaloric food alternatives for each meal as indicated during the initial counselling sessions. Portion sizes were standardised using visual aids and reference materials distributed at baseline. Composite indices representing dietary patterns (e.g. plant-based protein intake, healthy protein score) were computed post hoc based on frequency categories.

### Body composition and anthropometric measurements

Anthropometric measurements and body composition analysis were performed according to standardised protocols during the initial clinical visit, before starting the prescribed Mediterranean-type diet. Weight and body composition parameters were measured with a validated segmental body composition analyser (Tanita BC-420 MA), with participants in light clothing and fasting for at least 3 hours. Height was measured with a stadiometer using a standard anatomical alignment. WC was measured at the midpoint between the lower rib and iliac crest during minimal breathing. BMI was calculated as weight (kg) divided by height squared (m^2^).

ABSI was calculated using the formula: ABSI/1000 = WC/(BMI^⅔^ × height^½^) = WC weight-^⅔^ height^⅚^, as proposed by Krakauer and Krakauer (2012)^(9)^ to assess abdominal adiposity independent of BMI and height. Mean and SD ABSI by sex and age from the USA NHANES database were computed to calculate standardised dimensionless Z score on an individual basis^(9)^


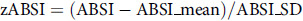




where ABSI_mean is the age and sex specific population mean and ABSI_SD is the SD. Spline smoothing was used to estimate ABSI_mean as a function of age, separately for men and women, and ABSI_SD was kept the same for all ages (since it showed no association with age) but was different for men and women.

### Exploratory indices

Several exploratory indices based on dietary and behavioural data were calculated, including the Mediterranean Pattern Score (MPS), Plant-Based Protein Score (PBPS) and Healthy Protein Score (HPS). These indices were derived post hoc using predefined cutoffs based on weekly food diary frequency data. In the final multivariable model, individual variables that showed significant associations in the adjusted models, such as nighttime consumption, playing a sport and taste preference, were included based on their predictive significance. Detailed formulas, scoring criteria and ranges for all composite indices are provided in the Supplementary Material (Description of Composite Scores). Behavioural variables such as ‘eat out’, ‘eat fast’, ‘eat alone’, ‘snacking between meals’ and ‘nighttime eating’ were assessed using a structured digital questionnaire previously developed at our centre and used in other studies by our research group. Each question included four predefined response options (‘Yes’, ‘No’, ‘Reluctantly’ and ‘Don’t know’). For analytical purposes, responses were dichotomised (Yes = 1; all other responses = 0). The detailed coding procedure is reported in the Supplementary Material (Behavioral Variable Coding).

### Statistical analysis

The primary outcome was the association between diet-related indices and *z*-standardised ABSI. Continuous variables were assessed for normality and summarised as mean ± sd. Categorical variables were reported as frequencies and percentages. Gender was included as an independent variable in all multivariable models to account for known physiological differences between males and females. Differences between males and females were tested using Welch’s t-test for continuous variables and chi-square test for categorical variables. Associations between dietary variables and ABSI were analysed using Pearson’s correlation coefficients. Composite dietary indices were also tested for correlation with ABSI and its standardised versions (*z*-scored ABSI (zABSI), also termed zABSI_internal because the standardisation was carried out using the study cohort’s mean and sd). Where appropriate, participants were divided into quartiles according to index values and one-way ANOVA was used to assess differences in ABSI measures between quartiles. Multivariable linear regression models were used to identify independent predictors of zABSI_internal. All analyses were conducted with Python (version 3.12), using libraries such as pandas, scipy and matplotlib. A *P*-value of less than 0·05 was considered statistically significant.

Multivariable linear regression models were then applied to identify independent predictors of zABSI_internal. Variables significantly associated with zABSI in age-, sex- and BMI-adjusted models (*P* < 0·05) were entered into the final model using an enter selection approach. Variance inflation factors (VIFs) were computed to assess multicollinearity, with all VIF values < 2·0, indicating no collinearity concerns. Model assumptions – including linearity, homoscedasticity and normality of residuals – were verified. Details on the model specification, selection strategy and diagnostic procedures are provided in the Supplementary Material (Multivariable Regression Model Specification).

## Results

The study included 1,640 adults, with a mean age of 41·5 ± 13·5 years; 42 % were males (*n* 694) and 58 % females (*n* 946). Males had significantly higher weight, BMI, fat mass and BMR than females (all *P* < 0·001), while females had significantly higher fat mass percentage and fat mass in kg (*P* < 0·001). The distribution of BMI categories is reported in Supplementary Table S1. Overall, 28·4 % of participants were of normal weight, 39·0 % were overweight and 32·0 % were living with obesity (21·2 % class I, 8·2 % class II and 2·6 % class III), while 0·7 % were underweight. The mean ABSI (expressed × 1000, unitless) was 92·9, slightly higher for men, whereas women had more variability in ABSI between individuals. Standardised ABSI scores (zABSI) had zero mean and unit sd for both men and women. The majority of participants reported performing <5 h of physical activity per week (69·7 %), with females reporting significantly less sports engagement than males (all *P* < 0·001). Table 1 shows the main features of the sample.

**Table 1.** Descriptive characteristics of the study population

	Total (*n* 1640)		Male (*n* 694)		Female (*n* 946)		
Variable	Mean	sd	Mean	sd	Mean	sd	*P*-value
Age (years)	41·5·	13·5	40·5·	13·4	42·2·	13·5	0·008
Weight (kg)	80·3·	17·7	89·8·	17·3	73·3·	14·5	<0·001
BMI (kg/m^2^)	28·2·	5·2	28·9·	5·1	27·7·	5·2	<0·001
Fat Mass (%)	30·8·	9·0	25·0·	7·5	35·0·	7·6	<0·001
Fat Mass (kg)	25·2·	10·7	23·4·	10·8	26·5·	10·4	<0·001
Fat-Free Mass (%)	65·8·	8·7	71·3·	7·3	61·8·	7·3	<0·001
Fat-Free Mass (kg)	52·3·	11·4	63·1·	8·3	44·5·	5·3	<0·001
ABSI	92·9·	5·6	93·5·	4·8	92·5·	6·1	<0·001
zABSI (unitless)	0·23·	0·98	0·33·	0·84	0·16·	1·06	<0·001
Abdominal Circumference (cm)	97·4·	14·2	101·9·	14·2	94·0·	13·2	<0·001
BMR (kcal/day)	1659·7·	347·4	1965·0·	277·8	1434·0·	182·3	<0·001
Current Smoker (%)	23·4 %		22·8 %		23·9 %		0·626
Sport participation (*n*, %)	876·	53·4 %	410·	59·1 %	466·	49·2 %	<0·001
Weekly sport participation among active individuals							
< 5 h	610·	69·7 %	246·	59·9 %	364·	77·9 %	<0·001
5–10 h	236·	27·0 %	144·	35·1 %	92·	20·2 %	<0·001
>10 h	30·	3·4 %	22·	5·2 %	8·	1·9 %	0·0075

Descriptive characteristics of the study population by sex. Continuous variables are presented as mean ± sd; categorical variables as percentages. *P*-values were calculated using Welch’s t-test for continuous variables and chi-square test for categorical variables. The body shape index (ABSI) was calculated as waist circumference divided by the product of BMI^(2/3)^ × height^(1/2)^ and is reported ×1000 for readability (dimensionless). The standardised ABSI (zABSI) was computed using NHANES reference values. Ranges for zABSI were –3.29 to 2.99 for the total sample, –2.79 to 2.71 for men, and –3.29 to 2.99 for women. Men had significantly higher zABSI values than women (*P* < 0.001), consistent with known sex differences in body shape indices.

The associations between zABSI and key dietary, behavioural and preference variables are summarised in Table 2. In univariate models adjusted for age, sex and BMI, several factors were significantly associated with zABSI. Participating in a sport showed a strong inverse association, while nighttime eating, snacking between meals and a preference for sweets were positively related to abdominal adiposity. Conversely, higher scores for healthy plant protein intake were inversely associated with zABSI. When all behavioural variables were included in the multivariate model, participation in sports and night-time eating remained significant predictors, which confirmed the protective role of regular physical activity and the negative association between night-time eating behaviour and central fat accumulation.

**Table 2.** Summary of univariate and multivariable associations between behavioural, dietary and preference variables and standardised ABSI (zABSI)

Variable	Direction of Association	*β* (Univariate)	*P*-value	*β* (Multivariable)*	*P*-value
Playing a sport	↓	−0·365	<0·001	−0·347	<0·001
Nighttime eating	↑	0·237	0·001	0·228	0·002
Snacking between meals	↑	0·133	0·014	NS	–
Skipping meals	↑	0·098	0·042	NS	–
Preference for sweet taste	↑	0·025	0·010	0·020	0·046
Preference for fruit	↓	−0·029	0·034	NS	–
Plant-Based Protein Score	↓	−0·040	0·002	–	–
Healthy Protein Score	↓	−0·028	0·004	–	–

**Figure 1.**
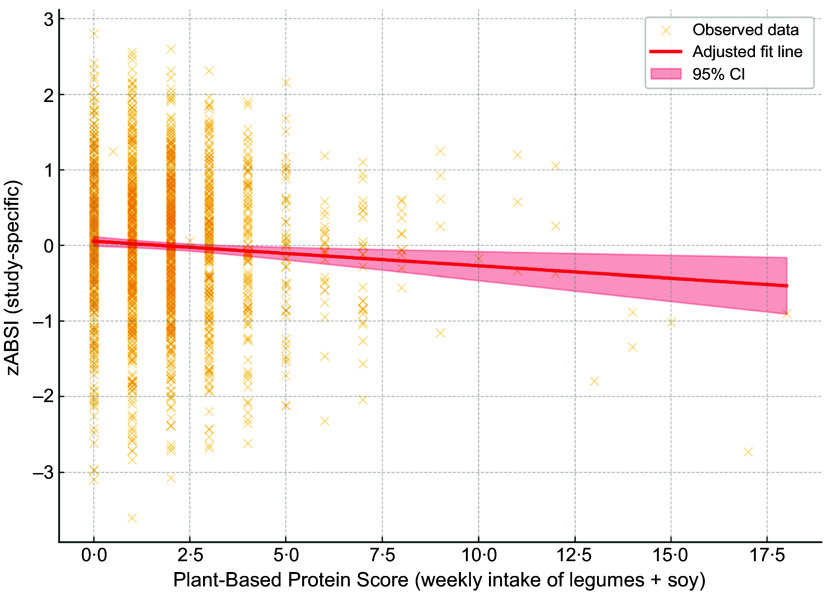
Association between zABSI and Plant-Based Protein Score. Adjusted linear regression plot showing the association between ABSI *z* score (zABSI) and Plant-Based Protein Score (weekly intake of legumes and soybeans), adjusted for age, sex and BMI. The red line represents the adjusted model, and the shaded area indicates the 95% confidence interval.

**Figure 2.**
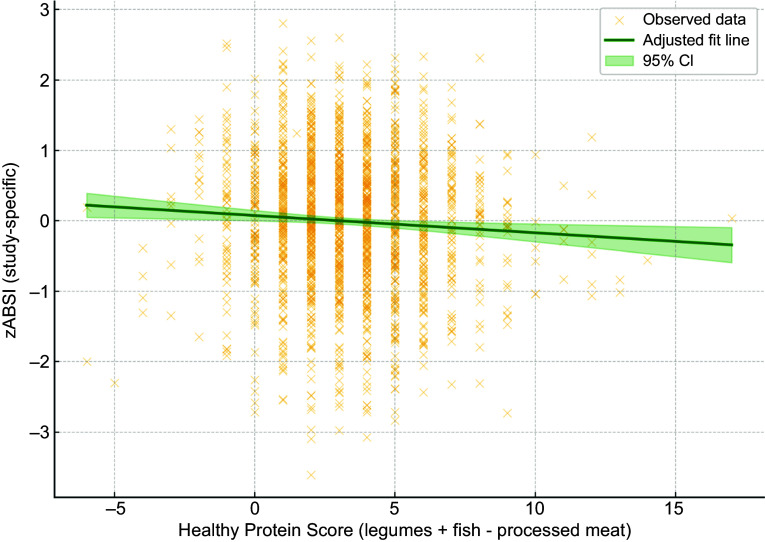
Association between zABSI and Healthy Protein Score. Adjusted linear regression plot showing the association between study-specific zABSI (zABSI_internal) and Healthy Protein Score (weekly intake of legumes + fish - processed meat), adjusted for age, sex and BMI. The green line represents the adjusted model, and the shaded area indicates the 95% confidence interval. The analysis demonstrates a significant inverse association (*β* = –0.021, *P* = 0.022).

**Figure 3.**
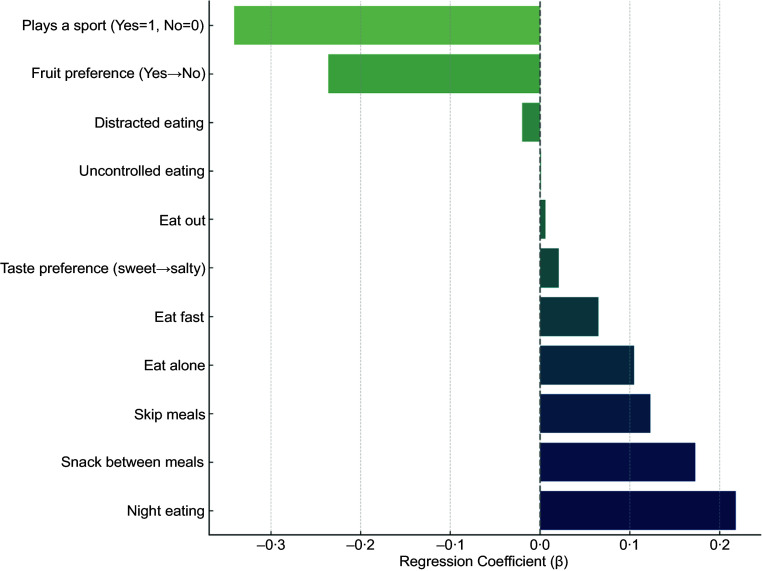
Adjusted associations from separate linear models for each variable (*n* 9), controlling for age, sex and BMI. Forest plot showing adjusted regression coefficients (*β*) and 95% confidence intervals for associations between zABSI and behavioural, taste, food preference and physical activity variables. All models were adjusted for age, sex and BMI.

**Figure 4.**
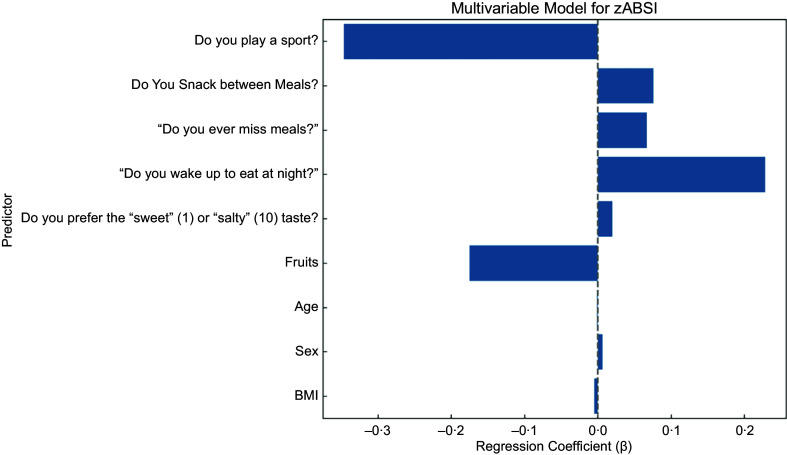
Multivariable linear model including all significant predictors jointly adjusted for age, sex and BMI.Forest plot of the adjusted regression coefficients (*β*) and 95% confidence intervals of the multivariable linear regression model predicting zABSI, an indicator of abdominal adiposity.

Summary of the associations between zABSI and the main behavioural and dietary predictors. The table integrates results previously shown in Figures 1–4 to improve clarity and allow easier interpretation. *Multivariable model adjusted for age, sex and BMI. ↓ inverse association with zABSI; ↑ positive association.

We examined the association between the ABSI *z*-score and the PBPS, which represents total weekly intake of legumes and soybeans. A linear regression model adjusted for age, sex and BMI revealed a statistically significant inverse association (*β* = –0·040, *P* = 0·002).

Similarly, we analysed the association between the study-specific *z*-scores of Body Shape Index A (zABSI_internal) and a newly constructed healthy protein score, defined as the sum of weekly legume and fish intake frequencies minus processed meat intake. After adjustment for age, sex and BMI, linear regression analysis revealed a significant inverse association (*β* = –0·028, *P* = 0·004).

In linear regression models adjusted for age, sex and BMI, several lifestyle, behaviour and preference variables were significantly associated with zABSI. Subjects who reported playing a sport had significantly lower zABSI values (*β* = –0·365, *P* < 0·001), suggesting a protective role of regular physical activity against abdominal adiposity. Other significant predictors were snacking between meals (*β* = 0·133, *P* = 0·014) and waking up at night to eat (*β* = 0·237, *P* = 0·001), which were positively associated with zABSI. In addition, preference for sweet taste over salty taste (*β* = +0·025 per unit on a 1–10 scale, *P* = 0·010) was associated with higher zABSI.

In the final multivariable model including behavioural factors as well as age, sex and BMI, playing a sport was most strongly associated with lower zABSI (*β* = –0·347, *P* < 0·001), underscoring the protective effect of regular physical activity. Waking up at night to eat remained significantly associated with higher internal zABSI (*β* = 0·228, *P* = 0·002). Preference for salty taste over sweet taste also remained significant (*β* = 0·020, *P* = 0·046). However, snacking between meals, skipping meals and preference for fruit did not maintain statistical significance in the multivariable model. These results suggest that the most consistent predictors of body shape variation are physical activity and nighttime eating, with taste preferences potentially also contributing. ABSI *z* score was not significantly associated with BMI, which supports the statistical independence of the two indices, and also not significantly associated with age and sex because of the age and sex specific means and standard deviations used to construct the *z* score.

## Discussion

In this large clinical cohort of adults who participated in a follow-up visit after a Mediterranean-type diet was prescribed, we explored the associations between diet quality, self-reported eating behaviours, food preferences and body shape using ABSI. ABSI is an allometric index that integrates WC, height and weight to describe body shape independently of BMI. It has been validated in numerous epidemiological studies as a predictor of mortality, cardiovascular events and metabolic dysfunction in different populations^(10–13)^. In contrast to BMI, which reflects general adiposity, ABSI specifically targets fat distribution, particularly visceral fat, making it a valuable tool for identifying individuals at high cardiometabolic risk who may not be considered by traditional anthropometric indices^(14,15)^. Recent literature has increasingly emphasised the role of fat distribution, rather than total fat mass, in the pathogenesis of metabolic disorders such as insulin resistance, non-alcoholic fatty liver disease and type 2 diabetes^(16,17)^. Central adiposity is metabolically more active and inflammatory than peripheral adiposity, which may explain the greater association of ABSI with adverse outcomes. The application of the age- and gender-standardised zABSI allows for finer risk stratification in the clinical setting and improves comparability between subgroups^(18)^. Despite these advantages, the relationship between ABSI and dietary patterns remains understudied. Most nutritional research continues to rely on BMI or WC as primary outcomes, while relatively few studies incorporate composite measures such as ABSI into dietary epidemiology. By incorporating the ABSI in a Mediterranean diet context, our study contributes to investigating how specific dietary components and behaviours – beyond total calorie intake or weight control – may influence fat distribution and cardiometabolic risk profiles^(19–21)^.

Waking up at night to eat was positively associated with higher zABSI values. This result is consistent with previous studies linking nocturnal food consumption to circadian disturbances, impaired glucose tolerance and visceral fat accumulation^(22–24)^. Nighttime food consumption can disturb the synchronisation between central and peripheral circadian clocks, leading to metabolic dysregulation. In particular, nocturnal biological food consumption may impair insulin sensitivity and glucose metabolism, promoting fat accumulation, especially in the abdominal region. Moreover, nocturnal food consumption has been associated with alterations in the expression of clock genes such as BMAL1, which plays a role in adipogenesis and lipid metabolism^(25)^. Overexpression of BMAL1 during nocturnal eating may increase lipid synthesis in adipose tissue, contributing to the accumulation of visceral fat. Furthermore, nocturnal eating behaviour may reduce melatonin secretion due to light exposure, further impairing glucose and lipid metabolism^(26)^.

The practice of sport was inversely associated with zABSI, indicating a potential protective role of physical activity against abdominal adiposity. This association is in line with existing literature suggesting that regular physical activity contributes to visceral fat reduction through various metabolic mechanisms^(27,28)^. One proposed mechanism is improved insulin sensitivity, which facilitates glucose uptake and reduces hyperinsulinaemia by inhibiting lipogenesis in visceral fat deposits. Physical activity also increases lipid oxidation, promoting the utilisation of fatty acids as an energy source. Furthermore, exercise influences adipokine profiles by increasing levels of adiponectin, an adipokine associated with anti-inflammatory effects and improved insulin sensitivity^(29)^. In addition, regular exercise decreases systemic inflammation by reducing pro-inflammatory cytokines such as TNF-α and IL-6, which are implicated in insulin resistance and central obesity.

The inverse association between HPS and zABSI observed in our study suggests that higher consumption of legumes and fish, combined with lower intake of processed meats, may favourably influence abdominal fat distribution through multiple biological pathways. Legumes, rich in fermentable fibre and resistant starch, promote the production of SCFAs by the gut microbiota, improving insulin sensitivity and reducing systemic inflammation^(30,31)^. Simultaneously, fish-derived omega-3 fatty acids (EPA and DHA) exert anti-inflammatory effects on adipose tissue, improve lipid metabolism and selectively reduce visceral fat accumulation^(32)^. In contrast, a high intake of processed meat is associated with increased oxidative stress, low-grade inflammation and impaired glucose metabolism^(33)^. These combined mechanisms support the hypothesis that specific protein sources, beyond total protein intake, play a crucial role in shaping body fat distribution, reinforcing the clinical relevance of promoting healthy protein choices to mitigate cardiometabolic risk.

Despite containing intrinsic sugars, fruit consumption has been consistently associated with favourable metabolic outcomes, including reduced visceral adiposity. Although fruit preference was not independently associated with zABSI in the multivariable model, it showed inverse associations in adjusted models and may still reflect healthier dietary habits. Mechanistically, fruit is rich in soluble fibre, which delays gastric emptying, reduces postprandial glucose excursions and increases satiety through modulation of gut hormones such as GLP-1 and PYY^(34)^. Furthermore, fruit is rich in polyphenols, particularly flavonoids, which exert anti-inflammatory and insulin-sensitising effects by activating AMP-activated protein kinase (AMPK) pathways and suppressing NF-κB-mediated inflammatory cascades^(35)^. These compounds inhibit adipogenesis and promote lipolysis in visceral fat deposits. Importantly, the fibre, water and bioactive matrix of whole fruits mitigate the metabolic impact of their sugar content, distinguishing them from refined sugars and processed foods. Prospective studies have shown that higher fruit intake correlates with reduced WC, lower ectopic fat deposition and improved insulin sensitivity, independent of total energy intake^(36)^. These data reinforce the concept that the overall nutritional profile of fruit – rather than its sugar content per se – determines its impact on body fat distribution. Promoting fruit consumption within Mediterranean dietary patterns may therefore be an effective strategy to mitigate central adiposity and reduce cardiometabolic risk.

Besides the quality of macronutrient sources, the timing and frequency of meals also emerged as relevant factors influencing the distribution of abdominal fat. Although meal skipping and snacking between meals showed positive associations with zABSI in univariate analyses, these associations did not persist in fully adjusted models. However, previous literature indicates that irregular dietary patterns, including skipping meals and frequent unhealthy snacking, may contribute to adverse metabolic effects and central fat accumulation. Skipping meals may alter circadian regulation of metabolism, impair glucose tolerance, increase postprandial insulin secretion and lead to compensatory overeating throughout the day^(37)^. In parallel, unhealthy snacking behaviours – especially energy-dense and nutrient-poor foods – have been linked to increased glycaemic variability, systemic inflammation and insulin resistance^(38)^. Mechanistically, irregular meal times can desynchronise peripheral metabolic clocks in tissues such as adipose tissue and liver, promoting lipogenesis and impairing lipid oxidation^(39)^. Although meal skipping and snacking between meals were initially significant predictors of zABSI in univariate models, their associations lost significance after multivariable adjustment. This attenuation is most likely due to confounding by broader lifestyle factors, particularly physical activity and nighttime eating, which remained significant and represent stronger correlates of abdominal adiposity in the full model. These behaviours tend to cluster: individuals with irregular eating patterns often also engage in less structured lifestyles, including lower physical activity levels, which can obscure the independent contribution of specific behaviours. A milder degree of multicollinearity among eating behaviour variables is also plausible, since meal skipping and snacking capture overlapping aspects of dietary irregularity. As a result, when entered together, their shared variance reduces the apparent individual effect of each variable. This interpretation is consistent with prior methodological literature emphasising how interrelated behavioural factors may lose statistical significance once modelled jointly^(40)^.

Despite the strengths of this study – including a large, well-characterised clinical sample and the use of validated anthropometric measures – some limitations must be acknowledged. Firstly, diet data were self-reported and collected only after a Mediterranean-type diet was prescribed, limiting causal inference and introducing potential signalling bias. Second, behavioural scores were based on customised questionnaire items, not validated psychometric instruments, and some variables had a high proportion of missing responses. Third, the cohort consisted of individuals who had adhered to follow-up visits and completed dietary records, likely introducing selection bias. Finally, the ABSI, while detecting abdominal adiposity, does not distinguish between visceral and subcutaneous fat compartments and its interpretation may vary between populations^(11)^. These limitations highlight the need to replicate the study in multi-ethnic prospective cohorts using validated instruments and biomarker data.

## Conclusions

In this large Mediterranean clinical cohort, we identified specific dietary and behavioural factors associated with body shape, as measured by a study-specific and BMI-independent zABSI. Our results show that nocturnal eating behaviour and low levels of physical activity are strongly associated with greater central fat accumulation. Although fruit preference was not independently associated with zABSI in the multivariable model, it showed inverse associations in adjusted models and may still reflect healthier dietary habits. These associations suggest that the timing, quality and behavioural context of diet may be as critical as macronutrient composition in shaping body fat patterns and metabolic risk. Our results emphasise the clinical utility of the ABSI as a complementary tool for abdominal adiposity assessment and cardiometabolic risk stratification in addition to traditional anthropometric measures. Future longitudinal studies and intervention trials are warranted to further elucidate the causal pathways underlying these associations and to inform the development of targeted, behaviour-focused nutritional strategies to mitigate central obesity and its complications.

## Supporting information

Lombardo et al. supplementary material 1 Lombardo et al. supplementary material

Lombardo et al. supplementary material 2 Lombardo et al. supplementary material

Lombardo et al. supplementary material 3 Lombardo et al. supplementary material

Lombardo et al. supplementary material 4 Lombardo et al. supplementary material

## Data Availability

The data supporting the findings of this study are available from the corresponding author upon reasonable request. All data will be shared in a de-identified format to protect participant confidentiality. This study was registered on ClinicalTrials.gov (NCT06654674).

## Supplementary material

To view supplementary material for this article, please visit https://doi.org/10.1017/S1368980025101729
