# Supplementary material for: Behavioral and dietary determinants of central adiposity assessed by ABSI in a mediterranean clinical sample
Source: Public Health Nutr. 2025 Dec 26;29(1):e10. doi: 10.1017/S1368980025101729 (PMC12895482; doi:10.1017/S1368980025101729)
Supplement: Lombardo et al. supplementary material 1 — Lombardo et al. supplementary material [file S1368980025101729sup001.docx]

# Supplementary Material – Behavioral Variable Coding

Behavioral data were obtained from the digital questionnaire administered at the first clinical visit. Each question offered four possible answers ('Yes', 'No', 'Reluctantly', and 'Don’t know') or equivalent descriptive options. For statistical analyses, variables were dichotomized as follows: Yes = 1 (behavior present); No/Reluctantly/Don’t know = 0 (behavior absent).

| Variable | Response categories in database | Recoding rule for analysis |
| --- | --- | --- |
| Do you ever miss meals? | Yes, No, Yes (breakfast only), Yes (snacking), Yes (no time) | Yes = 1; all others = 0 |
| Do you ever eat distracted or Not at the table? | Yes / No | Yes = 1; No = 0 |
| Do you eat fast? | Yes / No | Yes = 1; No = 0 |
| Do you eat out with meals? | Yes. I Eat at Restaurants, No | Yes = 1; No/empty = 0 |
| Do you eat alone or toghether? | Often Alone, Often Together, Alone at lunch | Alone = 1; Together = 0 |
| Do you happen to eat uncontrollably even if you're Not hungry? | Everyday, Often (>1/week), Rarely (1/month), Never | Everyday/Often = 1; Rarely/Never = 0 |
| Do You Snack between Meals? | Yes / No | Yes = 1; No = 0 |
| Do you wake up to eat at night? | Everyday, Often (>1/week), Rarely, Never | Everyday/Often = 1; Rarely/Never = 0 |
| How do you sleep at night? | Good, I struggle to fall asleep, I wake up several times, I wake up early | Analyzed qualitatively |
| Do you eat differently at the weekend? | Yes. I Eat at Restaurants, Yes. I Cook More Elaborate Meals, No | Yes = 1; No = 0 |
