# Supplementary material for: Behavioral and dietary determinants of central adiposity assessed by ABSI in a mediterranean clinical sample
Source: Public Health Nutr. 2025 Dec 26;29(1):e10. doi: 10.1017/S1368980025101729 (PMC12895482; doi:10.1017/S1368980025101729)
Supplement: Lombardo et al. supplementary material 2 — Lombardo et al. supplementary material [file S1368980025101729sup002.docx]

**Supplementary Material – Description of Composite Scores**

**Healthy Protein Score (HPS)**

The Healthy Protein Score was calculated as:

**HPS = (weekly servings of legumes + weekly servings of fish) – (weekly servings of processed meat)**

Higher scores indicate greater consumption of plant-based and fish protein sources relative to processed meat.

**Plant-Based Protein Score (PBPS)**

The Plant-Based Protein Score was based on total weekly intake of legumes and soy-derived foods (e.g., tofu, soy milk).

**PBPS = weekly servings of legumes + soy products**

Higher values represent stronger adherence to a plant-based protein pattern.

**Mediterranean Pattern Score (MPS)**

The Mediterranean Pattern Score was derived from 10 key food components typical of the Mediterranean diet (vegetables, fruits, whole grains, legumes, fish, olive oil, nuts, red meat, processed meat, and sweets). Each component was scored 1 point if the intake met the recommended frequency (≥5 servings/week for protective foods, ≤2 servings/week for limiting foods).

**MPS range: 0–10.** Higher scores indicate greater adherence to the Mediterranean dietary pattern.

All composite indices were computed from weekly frequency data collected through the 7-day food diary, reviewed by registered dietitians
