# Supplementary material for: Behavioral and dietary determinants of central adiposity assessed by ABSI in a mediterranean clinical sample
Source: Public Health Nutr. 2025 Dec 26;29(1):e10. doi: 10.1017/S1368980025101729 (PMC12895482; doi:10.1017/S1368980025101729)
Supplement: Lombardo et al. supplementary material 3 — Lombardo et al. supplementary material [file S1368980025101729sup003.docx]

**Multivariable Regression Model Specification**

- **Model type:** Multiple linear regression with standardized zABSI (internal) as the dependent variable.
- **Variable selection:** Independent variables were selected among those significantly associated with zABSI (p < 0.05) in adjusted univariate models controlling for age, sex, and BMI.
- **Entry method:** All selected variables were entered simultaneously (enter method) to assess independent contributions.
- **Confounder control:** Age, sex, and BMI were retained as covariates in all models.
- **Multicollinearity diagnostics:** Variance Inflation Factor (VIF) values < 2.0 for all predictors.
- **Model assumptions:** Linearity, homoscedasticity, and normality of residuals were checked using residual plots and Shapiro–Wilk tests.
- **Statistical software:** Python (version 3.12) with pandas, scipy, and statsmodels libraries.
