# Supplementary material for: Behavioral and dietary determinants of central adiposity assessed by ABSI in a mediterranean clinical sample
Source: Public Health Nutr. 2025 Dec 26;29(1):e10. doi: 10.1017/S1368980025101729 (PMC12895482; doi:10.1017/S1368980025101729)
Supplement: Lombardo et al. supplementary material 4 — Lombardo et al. supplementary material [file S1368980025101729sup004.docx]

### **Supplementary Table S1. Distribution of participants by BMI category (n = 1,640)**

| BMI Category | BMI Range (kg/m²) | n | % |
| --- | --- | --- | --- |
| Underweight | < 18.5 | 11 | 0.7 |
| Normal weight | 18.5–24.9 | 466 | 28.4 |
| Overweight | 25.0–29.9 | 640 | 39.0 |
| Obesity I | 30.0–34.9 | 347 | 21.2 |
| Obesity II | 35.0–39.9 | 134 | 8.2 |
| Obesity III | ≥ 40.0 | 42 | 2.6 |
